# A Case of Congenital Syphilis Diagnosed in an Infant Past the Newborn Period

**DOI:** 10.7759/cureus.44102

**Published:** 2023-08-25

**Authors:** Melanie D Vega, Rana Alissa, Mobeen Rathore

**Affiliations:** 1 Pediatric Infectious Diseases, University of Florida Center for HIV/AIDS Research, Education and Service (UF CARES), Jacksonville, USA; 2 Pediatric Infectious Diseases, Department of Pediatrics, University of Florida College of Medicine, Jacksonville, USA; 3 Pediatric Infectious Diseases, Wolfson Children’s Hospital/Baptist Health, Jacksonville, USA; 4 Pediatrics, Department of Pediatrics, University of Florida College of Medicine, Jacksonville, USA; 5 Pediatrics/Infectious Disease, University of Florida Center for HIV/AIDS Research, Education and Service (UF CARES), Jacksonville, USA

**Keywords:** nat vs syphilis, neonatal rash, neonatal fractures, endemic syphilis area, maternal syphilis testing, delayed diagnosis, congenital syphilis

## Abstract

This case report highlights the need for syphilis re-testing during pregnancy and at labor and delivery when there are high-risk factors present. Our patient, an infant, was evaluated for non-accidental trauma because of the presence of multiple fractures, which could be one of the presentations of congenital syphilis. A high index of suspicion is required for syphilis when an infant presents with multiple fractures. Syphilis testing and re-testing guidelines should be followed strictly so that pregnant women are appropriately treated to prevent congenital syphilis.

## Introduction

Rates of congenital syphilis have been steadily rising and have more than tripled in recent years [1]. Congenital syphilis is often asymptomatic but can also result in preterm birth and hydrops fetalis [2]. A patient can present with snuffles, maculopapular rash with spots on the palms and soles, hepatosplenomegaly, osteochondritis, hemolysis, and pseudoparalysis within two months of birth [2]. In addition, untreated syphilis in pregnant women is a common cause of stillbirth and neonatal death [3]. The Centers for Disease Control and Prevention (CDC) recommends testing all pregnant women at the time of their first prenatal visit and at 28 weeks of gestation for those women at risk for infection or re-infection [1]. In addition, for some high-risk women who have tested negative, an additional test for syphilis is recommended at the time of labor and delivery [1]. In the state of Florida, by administrative rule, it is recommended that all pregnant women be tested for syphilis at their initial visit related to their pregnancy, again between 28 and 32 weeks of gestation, and at the time of labor and delivery if they are at high risk for the infection. In addition, women who present at delivery with no documented syphilis test should be tested for syphilis [4].

We report a case of an infant diagnosed with congenital syphilis past the newborn period, while the mother had negative prenatal testing for syphilis early in pregnancy.

## Case presentation

**Figure 1:**
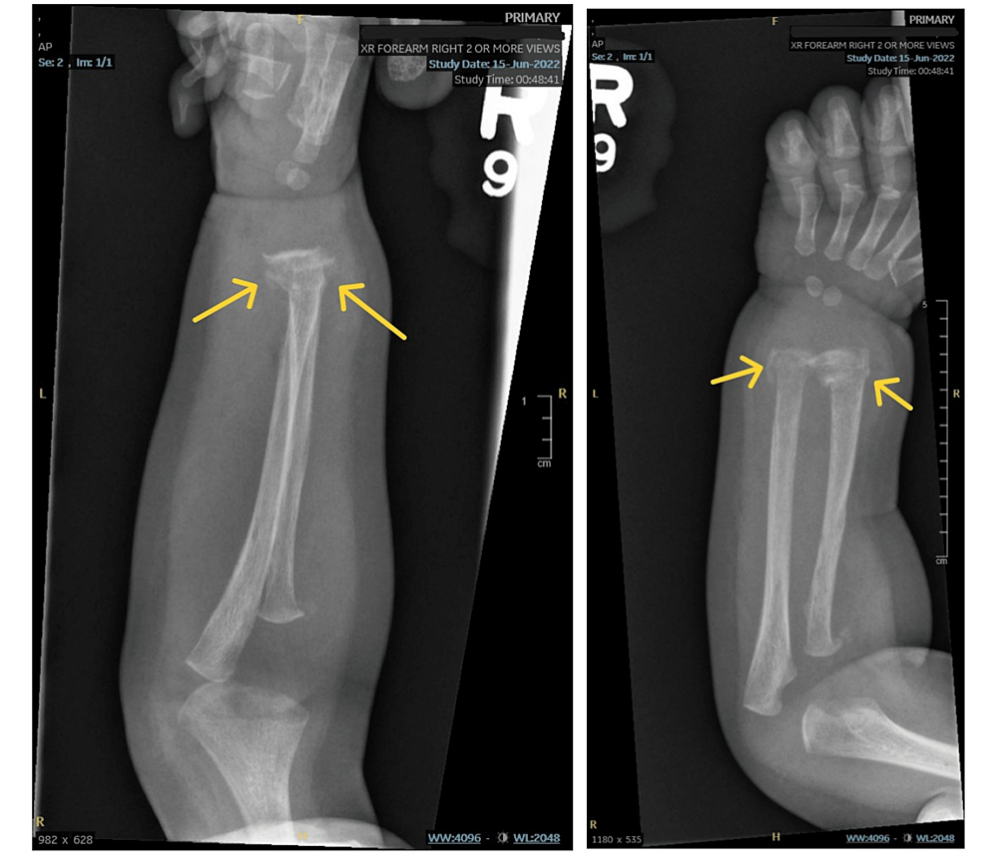
X-rays of healing fractures in the distal radius and ulna on initial presentation

A seven-week-old female born at 39 weeks of gestation via elective repeat C-section presented to our pediatric emergency room with a right arm deformity. The syphilis screen was non-reactive, and the group B strep (GBS) screen was positive during the third trimester. Screens for hepatitis B surface antigen and gonorrhea were negative in the mother at 32 weeks of gestation. At 36 weeks of gestation, tests for *Chlamydia trachomatis* and *Trichomonas vaginalis* were positive, and these infections were treated. The mother was homeless and had a history of marijuana use. On examination, the baby was not moving the right wrist much; it was swollen and felt warm. The mother at the time did not perceive that the patient had any pain and denied any history of trauma. The patient was evaluated at another hospital earlier in the day, where an X-ray showed a fracture of the distal radius and ulna (Figure 1).

**Figure 2:**
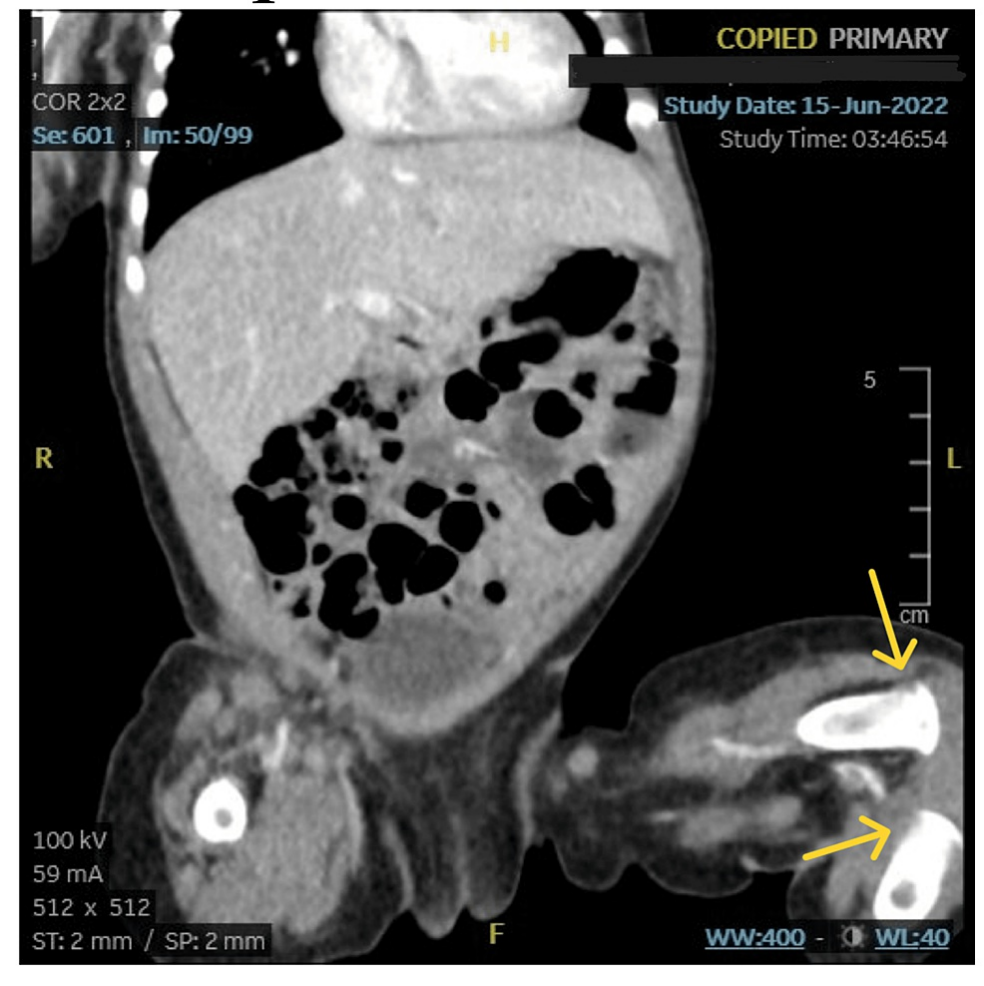
A CT scan of the abdomen revealed distal tibial and femoral fractures.

The X-ray also showed old fractures in the radial shaft and neck. The patient was transferred to our facility on suspicion of non-accidental trauma (NAT). At the time of evaluation at our hospital, in addition to what was reported previously, the patient had 5 mm to 6 mm discolored skin lesions on the lower extremities. The rest of the examination was unremarkable. Laboratory evaluation showed hemoglobin of 6.3 g/dL, hematocrit of 18.8%, alanine transaminase (ALT) of 56 U/L, and aspartate aminotransferase (AST) of 54 U/L. A respiratory viral panel was positive for parainfluenza type 3. A CT scan of the abdomen and pelvis was normal except for subtle metaphyseal lucencies, which were consistent with distal tibial and femoral fractures (Figure 2).

The CT scan of the head was normal. A skeletal survey showed multiple metaphyseal fractures in the upper and lower extremities. The patient’s right limb was immobilized, and she received a unit of packed red blood cells. Evaluation for osteogenesis imperfecta (OI) was commenced. Because of concern for NAT, the patient was discharged on the fourth day of hospitalization in the custody of the grandmother with supervised visitation by the mother. A follow-up appointment was scheduled with the consulting orthopedic surgeon.

**Figure 3:**
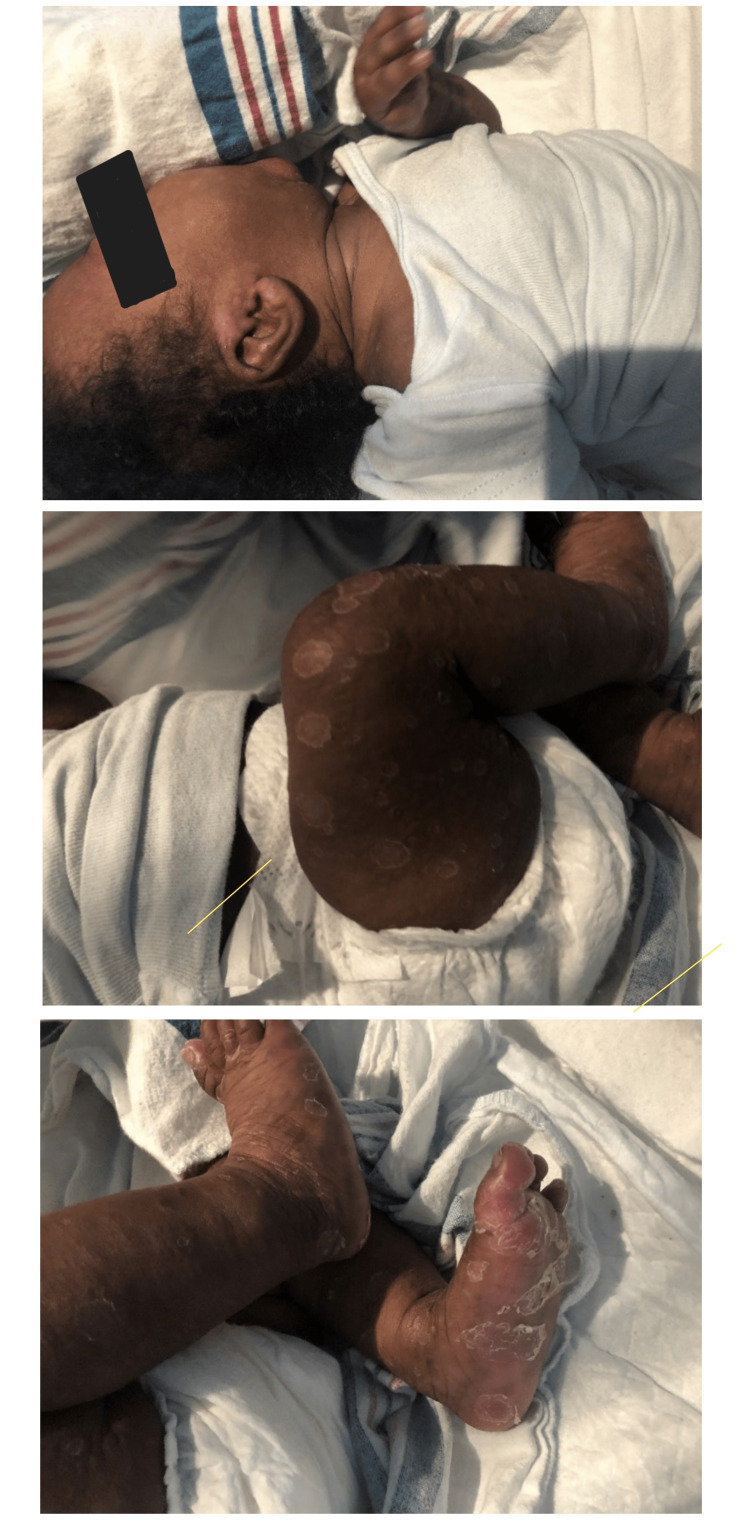
The rash that started on the patient's face spread to the lower extremities.

The patient returned to the emergency department (ED) nine days after discharge with a history of fever and a worsening skin rash. The rash had spread from the face to the extremities, including the palms and soles. The rash consisted of multiple 1-2 cm target-like raised wheals with varying degrees of pigmentation and was desquamating (Figures 3A-3C).

**Figure 4:**
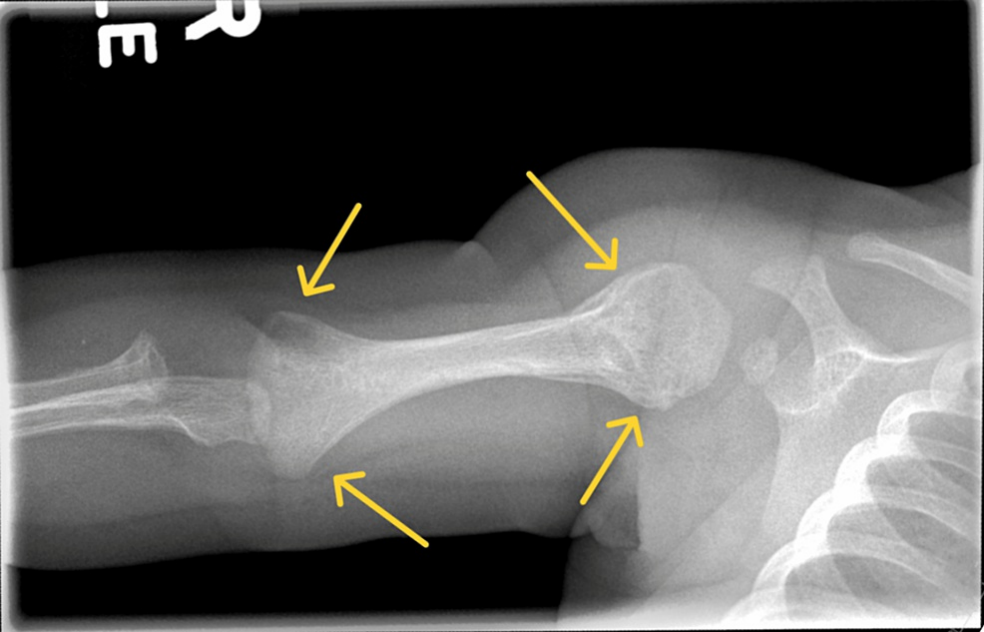
A repeat skeletal survey showed healing fractures of the distal left humeri.

At this visit, the mother also had a rash on her palms and soles, which was mildly pruritic but not painful. When informed of plans for admission and intravenous (IV) antibiotics, the mother and grandmother left against medical advice. The grandmother brought the baby back to the ED the next day. The mother was incarcerated at this time for unrelated reasons. A repeat skeletal survey showed healing fractures of the proximal right and distal left humeri but no new fractures (Figure 4).

Further healing of multiple metaphyseal fractures of the upper and lower extremities was also noted. No retinal hemorrhages were present, and a CT of the head was normal again. Osteogenesis imperfecta was ruled out by this time. At this time, because of the rash, syphilis was suspected. A serum rapid plasma reagin (RPR) test was performed, which was reactive at 1:512. The cerebrospinal fluid (CSF) venereal disease research laboratory (VDRL) test was also reactive. Since her mother had not been tested for syphilis since the third trimester (29 weeks of gestation), she was offered a syphilis test, which she declined.

The patient was treated with IV penicillin (50,000 units/kg) every eight hours for a total of 14 days. The patient was discharged with follow-up with orthopedics, physical therapy, and pediatric infectious disease for repeat testing after treatment. The patient did not return for pediatric infectious disease follow-up after treatment. She was followed by occupational therapy services and was discharged since she was meeting all developmental milestones.

## Discussion

**Figure 5:**
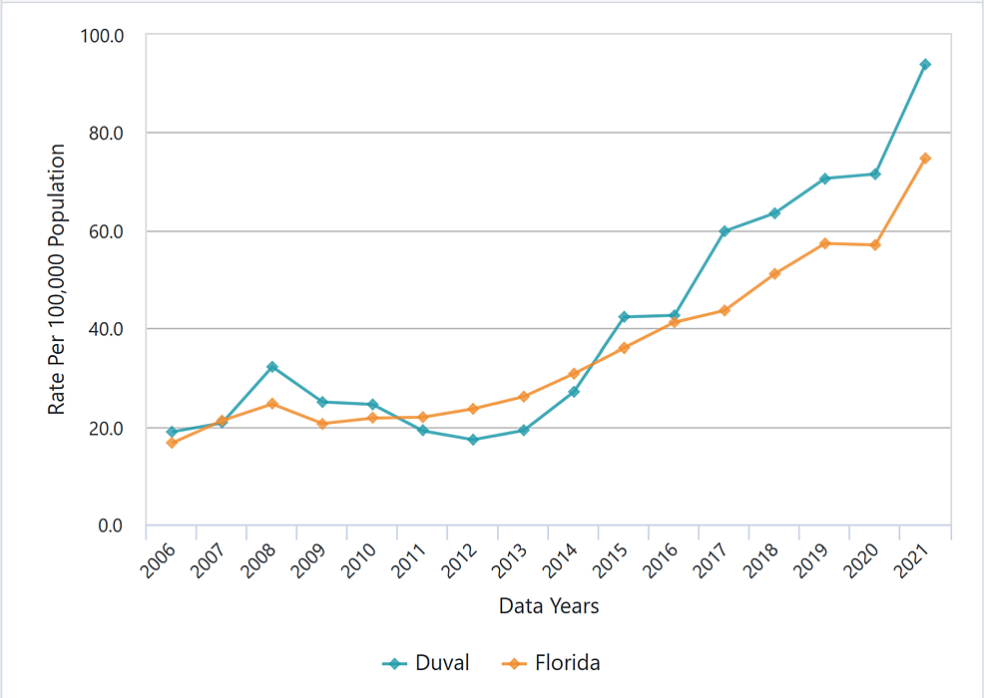
Syphilis cases in Florida and Duval County Source: Florida Department of Health [5]

**Figure 6:**
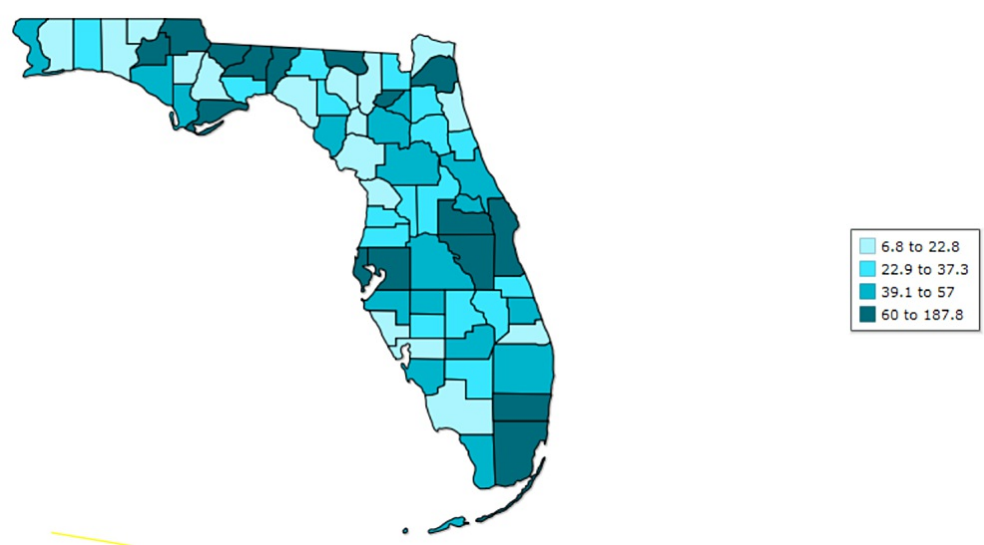
Syphilis, all stages, rate per 100,000 population, 2021 Duvall County Source: Florida Department of Health [5]

**Figure 7:**
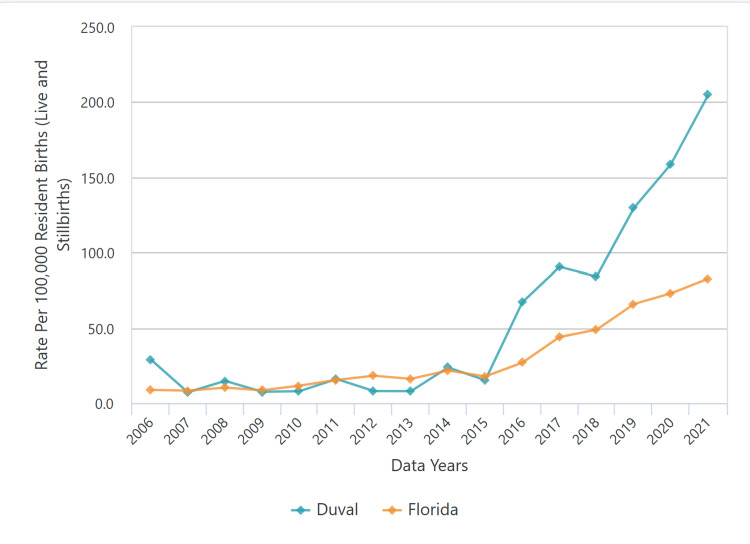
Congenital syphilis cases in Florida and Duval County Source: Florida Department of Health [6]

Syphilis is a sexually transmitted disease caused by the spirochete *Treponema pallidum*. Newborns acquire the infection transplacentally or perinatally due to contact with infected maternal lesions [2]. Congenital syphilis is defined as early if clinical manifestations appear before the age of two and late if symptoms occur at two years of age or older [4]. Both CDC guidelines and state regulations, although slightly different, are structured to identify women who may be infected with syphilis during pregnancy, even if they test negative earlier in pregnancy. Some of the high-risk conditions that are indications for repeat testing for syphilis at 28 weeks of gestation and during labor and delivery include women who live in communities with high syphilis rates or those who have other risk factors for the acquisition of syphilis during pregnancy, including misuse of drugs, sexually transmitted infections (STIs) during pregnancy, multiple sex partners, having a new sex partner, a sex partner with an STI, late entry into or no prenatal care, incarceration of women or her partner, and unstable housing or homelessness. Our patient’s mother had several of these risk factors. In addition, Florida, and especially Duval County in northeast Florida, has faced an exponential increase in cases of syphilis (Figures 5-6) and, not surprisingly, a parallel increase in cases of congenital syphilis (Figure 7).

Our area is considered an endemic area, with one of the highest rates of syphilis in the state of Florida [7].

Congenital syphilis is a preventable and treatable disease. However, screening for syphilis must be done in a timely manner, and providers must be aware of the prevalence of syphilis in their area. The crucial elements of maternal history-both infection and treatment, as well as physical examination-are the key to reaching the correct diagnosis. In our case, the mother was extremely high-risk due to homelessness, a history of STIs, and incarceration, which are all indications for testing at the time of delivery [3]. Additionally, the patient had several manifestations of congenital syphilis, including anemia, pseudoparalysis, numerous fractures, and a rash. It should also be noted that the mother had a maculopapular rash on her palms. Finally, the fractures led to a focus on NAT versus consideration of other possible conditions, such as congenital syphilis. This case highlights the importance of repeated screening for syphilis during pregnancy and at the time of delivery for pregnant women who have high-risk factors, including those residing in high-risk endemic areas. There should be a high index of suspicion for syphilis in any newborn, especially if the mother meets the high-risk criteria set by the CDC for syphilis screening.

A recent increase in congenital syphilis rates has been reported in the US [1, 7, 8, 9]. Many experts consider the rising rates a public health problem. In 2019, about 60% of congenital syphilis cases were due to gaps in testing and treatment during prenatal care, especially those related to no prenatal care [7]. Factors such as poverty, lack of healthcare, and low sexual health literacy play a role in the low rates of testing and treating syphilis during pregnancy [7]. Even internationally, namely in Brazil, where the WHO has initiatives to decrease HIV and syphilis mother-to-child transmission, congenital syphilis cases continue to increase [10]. As with our case, the testing and treatment of the mother cannot be overemphasized. The prevalence of syphilis in the area should also be considered.

## Conclusions

This case also illustrated how one of the multitude of clinical manifestations of congenital syphilis can make early diagnosis a challenge if there is not a high index of suspicion. Congenital syphilis should be considered in the differential diagnosis of pediatric patients in the first two years of life presenting with fractures or rashes consistent with syphilis.
